# Roles of the crotonyl-CoA carboxylase/reductase homologues in acetate assimilation and biosynthesis of immunosuppressant FK506 in *Streptomyces tsukubaensis*

**DOI:** 10.1186/s12934-015-0352-z

**Published:** 2015-10-14

**Authors:** Marko Blažič, Gregor Kosec, Špela Baebler, Kristina Gruden, Hrvoje Petković

**Affiliations:** Department of Food Science and Technology, Biotechnical Faculty, University of Ljubljana, Jamnikarjeva 101, 1000 Ljubljana, Slovenia; Acies Bio, d.o.o., Tehnološki park 21, 1000 Ljubljana, Slovenia; Department of Biotechnology and Systems Biology, National Institute of Biology, Večna pot 111, 1000 Ljubljana, Slovenia

**Keywords:** *Streptomyces tsukubaensis*, FK506, Tacrolimus, Extender units, Ethylmalonyl-CoA, Allylmalonyl-CoA, Crotonyl-CoA carboxylase/reductase (*ccr*), Ethylmalonyl-CoA pathway (EMC), Polyketide, Polyketide synthase (PKS), Acetate assimilation

## Abstract

**Background:**

In microorganisms lacking a functional glyoxylate cycle, acetate can be assimilated by alternative pathways of carbon metabolism such as the ethylmalonyl-CoA (EMC) pathway. Among the enzymes converting CoA-esters of the EMC pathway, there is a unique carboxylase that reductively carboxylates crotonyl-CoA, crotonyl-CoA carboxylase/reductase (Ccr). In addition to the EMC pathway, gene homologues of *ccr* can be found in secondary metabolite gene clusters that are involved in the provision of structurally diverse extender units used in the biosynthesis of polyketide natural products. The roles of multiple *ccr* homologues in the same genome and their potential interactions in primary and secondary metabolic pathways are poorly understood.

**Results:**

In the genome of *S. tsukubaensis* we have identified two *ccr* homologues; *ccr1* is located in the putative ethylmalonyl-CoA (*emc*) operon and *allR* is located on the left fringe of the FK506 cluster. AllR provides an unusual extender unit allylmalonyl-CoA (ALL) for the biosynthesis of FK506 and potentially also ethylmalonyl-CoA for the related compound FK520. We have demonstrated that in *S. tsukubaensis* the *ccr1* gene does not have a significant role in the biosynthesis of FK506 or FK520 when cultivated on carbohydrate-based media. However, when overexpressed under the control of a strong constitutive promoter, *ccr1* can take part in the biosynthesis of ethylmalonyl-CoA and thereby FK520, but not FK506. In contrast, if *ccr1* is inactivated, *allR* is not able to sustain a functional ethylmalonyl-CoA pathway (EMC) and cannot support growth on acetate as the sole carbon source, even when constitutively expressed in the chimeric *emc* operon. This is somewhat surprising considering that the same chimeric *emc* operon results in production of FK506 as well as FK520, consistent with the previously proposed relaxed specificity of AllR for C4 and C5 substrates.

**Conclusions:**

Different regulation of the expression of both *ccr* genes, *ccr1* and *allR*, and their corresponding pathways EMC and ALL, respectively, in combination with the different enzymatic properties of the Ccr1 and AllR enzymes, determine an almost exclusive role of *ccr1* in the EMC pathway in *S. tsukubaensis*, and an exclusive role of *allR* in the biosynthesis of FK506/FK520, thus separating the functional roles of these two genes between the primary and secondary metabolic pathways.

**Electronic supplementary material:**

The online version of this article (doi:10.1186/s12934-015-0352-z) contains supplementary material, which is available to authorized users.

## Background

**Fig. 1:**
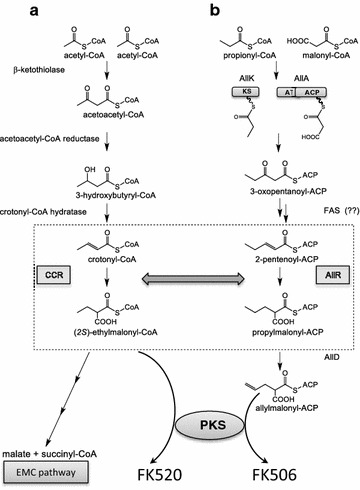
Schematic presentation of the EMC pathway providing ethylmalonyl-CoA extender unit (**a**) and of the proposed ALL pathway (**b**) for biosynthesis of allylmalonyl-CoA extender unit, part of the FK506 biosynthetic machinery. The step of potential cross-activity of *ccr* homologues from EMC and ALL pathways is marked by a two-way *arrow*

In microorganisms that can assimilate acetate, but lack a functional glyoxylate cycle (GLC) [1], the recently identified ethylmalonyl-CoA pathway (EMC) [2] is used as an alternative pathway in carbon metabolism. Genes that are involved in this pathway have been identified in the genomes of many bacteria including many α-proteobacteria, like *Rhodobacter sphaeroides* and *Methylobacterium extorquens,* as well as in diverse actinomycete species [3]. In contrast to the GLC cycle, the EMC pathway contains many unique CoA-ester intermediates, such as (2R)- and (2S)-ethylmalonyl-CoA, (2S)-methylsuccinyl-CoA, mesaconyl-(C1)-CoA, and (2R,3S)-methylmalyl-CoA (Fig. 1). Among the enzymes converting these CoA-esters there is a unique carboxylase that reductively carboxylates crotonyl-CoA, crotonyl-CoA carboxylase/reductase (Ccr) [2]. This step is followed by reactions catalysed by ethylmalonyl-CoA epimerase, ethylmalonyl-CoA mutase and methylsuccinyl-CoA dehydrogenase, the biosynthetic steps which are considered as key steps for the assimilation of acetyl-CoA through the EMC pathway [2]. As a consequence of the EMC pathway, net succinyl-CoA and malate are generated from three molecules of acetyl-CoA, one molecule of CO_2_ and one molecule of HCO_3_^−^ (Fig. 1a).

**Fig. 2:**
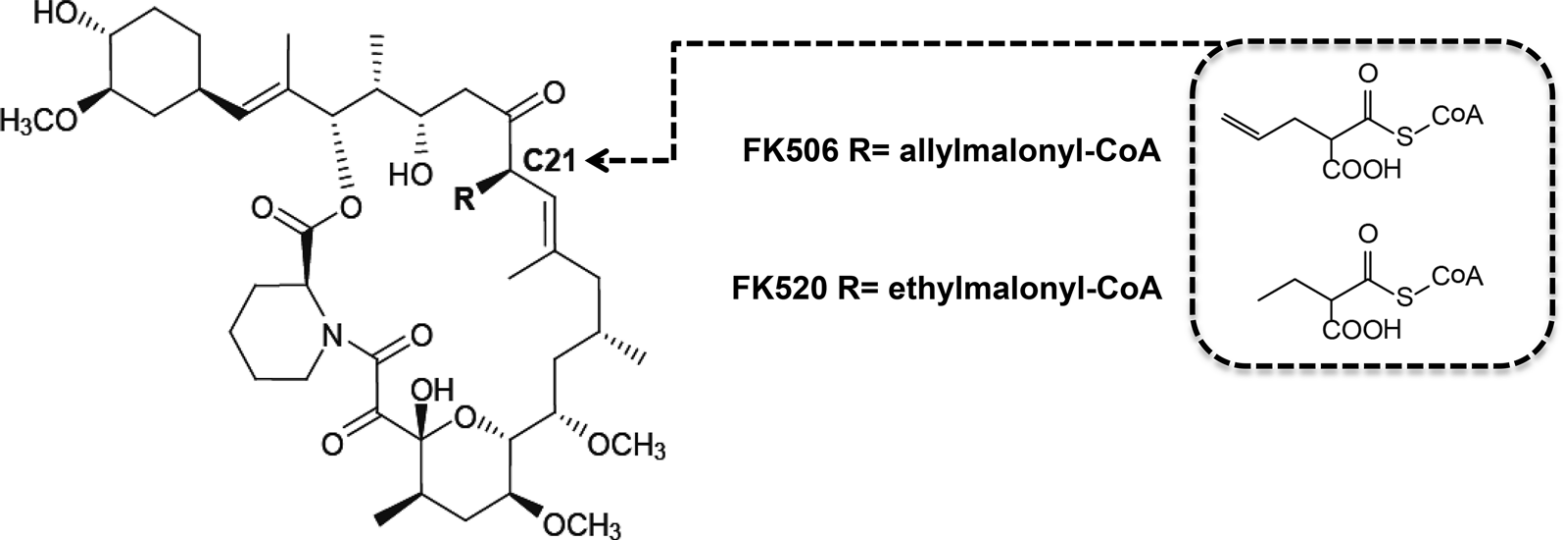
The structures of FK506 and FK520 differ only in the side chain moiety at the carbon C21. Incorporation of the allylmalonyl-CoA and ethylmalonyl-CoA extender unit analogue into the structure of FK506 is marked in the *box*

Core genes related to the EMC pathway have been found in the genomes of many species belonging to the order *Actinomycetales*. They are in general localized together, possibly in a single operon (referred here as the “*emc* operon”) [3]. Interestingly, besides the *emc* operon, additional copies of gene homologues of crotonyl-CoA carboxylase/reductase (*ccr*) can be found in numerous gene clusters encoding for the biosynthesis of secondary metabolites [3]. Secondary metabolites of polyketide origin most often incorporate extender units malonyl-CoA and methylmalonyl-CoA. However, the literature describes numerous polyketides with side chains that cannot be explained retrobiosynthetically, when taking into account only the precursors that originate from primary metabolic pathways. It has been demonstrated that in many secondary metabolite gene clusters *ccr* homologues are present and play essential roles in the biosynthesis of unusual extender units, such as ethylmalonyl-CoA. These homologues are involved in the reductive carboxylation of diverse 2-alkenoyl carboxylic acids, resulting in the formation of unusual alkylmalonyl thioester building blocks, used in the biosynthesis of the polyketide backbone (Fig. 1b), thus expanding the structural diversity of these biologically active metabolites [4]. For example, *ccr* homologues were identified in gene clusters encoding for the biosynthesis of FK506 (tacrolimus) and FK520 (ascomycin), which are produced by the industrially used strains *Streptomyces tsukubaensis* and *Streptomyces hygroscopicus var. ascomyceticus*, respectively [5]. The biosynthesis of FK506 and FK520 by *S. tsukubaensis*, for example, demands simultaneous availability of at least 6 precursors from diverse metabolic pathways, which have to be expressed simultaneously during the production of FK506 [6–8]. Biosynthesis is carried out by a polyketide synthase/non-ribosomal peptide synthetase enzyme complex (PKS/NRPS) in a highly coordinated action. The structures of FK506 and FK520 differ only in a single moiety located at the carbon C21 position (Fig. 2). FK520, which represents an undesired impurity in the industrial production of the medically important immunosuppressant FK506, is formed as a result of the incorporation of ethylmalonyl-CoA instead of a allylmalonyl-CoA extender unit, due to the promiscuous nature of the acyltransferase (AT) domain of the module 4 of FK506 PKS [5, 9, 10]. As a consequence, an ethyl moiety, instead of an allyl group, is present at the carbon C21 position in the polyketide backbone, thus resulting in FK520 (Fig. 2). While the pathway providing the allylmalonyl-CoA extender unit has been identified [5, 9], the biosynthetic origin of the ethylmalonyl-CoA extender unit in FK506-producing *Streptomyces* strains has not yet been clearly elucidated. The formation of FK520 during the industrial production of the medically important immunosuppressant FK506 represents a significant financial and environmental burden, since this impurity is technically very difficult to remove during chromatographic purification steps [11]. Considering that the biosynthetic origin of ethylmalonyl-CoA in *S.tsukubaensis* is still not well understood and it can potentially have multiple sources, it was our aim to investigate the possible cross-activity between *ccr* homologues from the *emc* operon (designated as *ccr1*) and *allR*, a *ccr* homologue located in the FK506 gene cluster (Fig. 1). Furthermore, our aim was to assess the substrate specificity of these two *ccr* homologues in vivo and their potential role in primary metabolic pathways such as acetate assimilation.

In the scope of this work, we have clearly demonstrated that the EMC pathway plays an important role in acetate assimilation in *Streptomyces tsukubaensis* NRRL 18488; however, it is not significantly expressed in standard carbohydrate-based growth media. Therefore, in *S. tsukubaensis,* the enzymes of the EMC pathway do not supply a significant flow of ethylmalonyl-CoA to the FK506-PKS biosynthetic machinery, at least not in the media used for the efficient industrial production of FK506. This result suggests a complex regulation of the expression of the enzymes of the EMC pathway, in accordance with its primary functional role dedicated to the assimilation of acetate in the scope of primary metabolism.

## Results

*Streptomyces tsukubaensis* NRRL 18488 belongs to the group of microorganisms that contain the EMC pathway, as clearly demonstrated by genome analysis carried out in the scope of this work [12–14]. As expected, the putative *emc* operon from *S. tsukubaensis* has the typical gene organisation found in other *Streptomyces* species (Additional file 1: Figure S1) [3] and it contains a crotonyl-CoA carboxylase/reductase homologue (designated as *ccr1*) (Fig. 1a). Interestingly, the second *ccr* gene homologue present in *S. tsukubaensis* (designated as *allR* [5]) is located in the small operon on the left fringe of the FK506 gene cluster, encoding for the biosynthesis of an allylmalonyl-CoA extender unit. The amino acid sequence of AllR shows a very high similarity (61 % identity and 74 % similarity) to that of *ccr1* (Additional file 2: Figure S2).

### Analysis of the expression of the crotonyl-reductase/carboxylase homologues *ccr1* and *allR* using qPCR

**Fig. 3:**
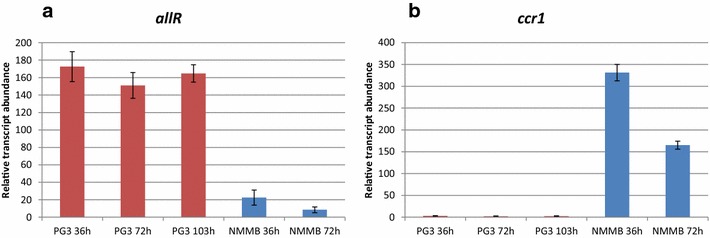
Expression levels of *ccr1* gene from EMC pathway and *allR* gene from FK506 gene cluster in NMMB medium supplemented with acetate a sole source of carbon (*blue bars*) and in the complex medium PG3, supporting FK506 production (*red bars*)

In order to evaluate the expression profiles of both *ccr* homologues we conducted qPCR analyses. To assess the expression of the *emc* operon, we cultivated *S. tsukubaensis* in a minimal medium based on NMMB, solely containing acetate as a source of carbon [15]. Alternatively, in order to detect the expression of the *allR* gene, the complex glucose/dextrin—containing medium PG3 was used for cultivation, as FK506 is produced at a good yield in this medium. Total RNA was isolated from *S. tsukubaensis* cultures following 36, 72 and 103 h of cultivation in PG3 medium; 36 and 72 h time points were established as the optimal times for the analysis in minimal medium, the cultivation time being much shorter due to lower amounts of the available carbon source. The results clearly showed that *ccr1* was expressed in the minimal medium NMMB supplemented with acetate (Fig. 3). In contrast, expression of *ccr1* was below the limit of quantification in the FK506 production medium PG3. Interestingly, the expression of the *allR* homologue from the FK506 gene cluster was detected in both media. Although expression of *allR* was detected in cultures grown in NMMB minimal medium supplemented with acetate, its levels were less than 10 % of those in cultures grown in the glucose/dextrin-based medium PG3 (Fig. 3).

### The role of crotonyl-CoA reductase/carboxylase homologues *ccr1* and *allR* in acetate assimilation

**Fig. 4:**
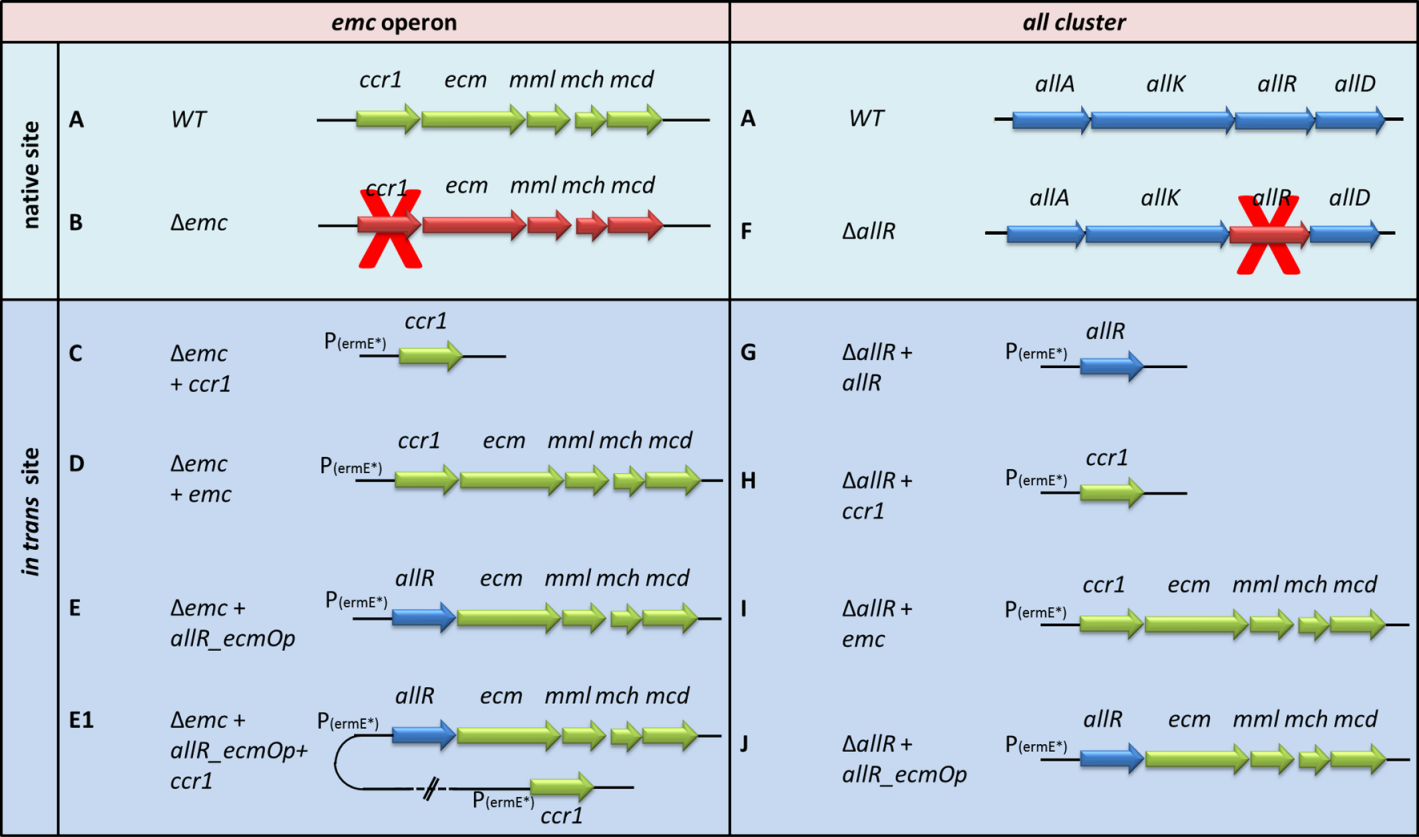
Schematic presentation of *emc* operon and *all* group of genes and corresponding constructs used in this study. Strain: WT strain (**a**), *Δemc* (**b**), *Δemc* + *ccr1* (**c**), *Δemc* + *emc* (**d**), *Δemc* + *allR_ecmOp* (**e**), *Δemc* + *allR_ecmOp* + *ccr1* (**e**1), *ΔallR* (**f**, [11]), *ΔallR* + *allR* (**g**), *ΔallR* + *ccr1* (**h**), *ΔallR* + *emc* (**i**) and *ΔallR* + *allR_ecmOp* (**j**)

**Fig. 5:**
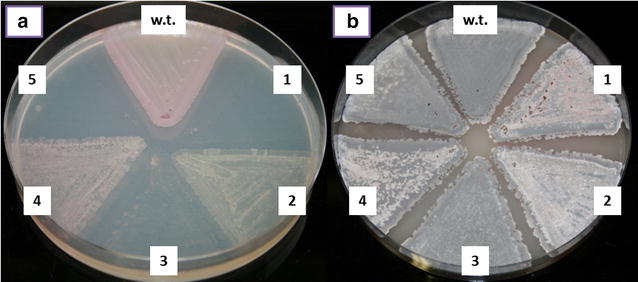
Growth of various mutants of *S. tsukubaensis* on the minimal medium (**a**) in which the acetate is present as a sole carbon source and on complex medium ISP4 (**b**). Strains WT (*S. tsukubaensis* NRRL 18488); *1* *Δemc* (**b**); *2* *Δemc* + *emc* (**d**); *3 Δemc* + *allR_ecmOp* (**e**); *4* *Δemc* + *allR_ecmOp* + *ccr1* (**e**1); *5* Δemc + ccr1 (**c**). *Letters in brackets* correspond to the labels of the constructs presented on Fig. 4

In the next step, our aim was to determine the functional role of the crotonyl-CoA reductase/carboxylase homologues *ccr1* and *allR* in relation to acetate assimilation. For this purpose we constructed two engineered strains of *S. tsukubaensis*. In one strain the *allR* gene, located in the FK506 gene cluster, was deleted *in*-*frame* (designated as *S. tsukubaensisΔallR* (Fig. 4f, [11]) and in the second strain, designated as *S. tsukubaensisΔemc*, the *ccr1* gene was disrupted, causing the inactivation of the entire *emc* operon (Fig. 4b, [11]). Complementation experiments were carried out by the *in*-*trans* complementation approach where different plasmid constructs containing combinations of *ccr1*/*allR* genes and the remaining *emc* operon genes were expressed constitutively in the two gene-inactivated strains (See Materials and Methods section) (Fig. 4c–j). Engineered strains were tested for their ability to grow on minimal NMMB agar medium supplemented with acetate as the sole carbon source (Fig. 5a). In a parallel control experiment, the same strains were cultivated on the nutrient-rich sporulation medium ISP4 [16], where no significant differences in growth or sporulation intensity were observed (Fig. 5b). As expected, inactivation of *allR* did not affect the growth of the strain on the minimal medium (result not shown). In contrast, inactivation of the EMC pathway through the disruption of *ccr1* (strain *Δemc* (B)) resulted in a complete loss of the ability of *S. tsukubaensis* to grow on acetate as the sole source of carbon (Fig. 5a1). As expected, *in*-*trans* complementation of the *Δemc* strain with the *ccr1* gene alone (*Δemc* + *ccr1)* failed to restore growth on acetate as the sole carbon source (Fig. 5a5), further indicating polycystronic transcription of the genes of the *emc* operon. In contrast, when the *Δemc* strain was complemented by the entire wild type (native) *emc* operon [*Δemc* + *emc* (Fig. 4d)] under the control of the ermE* promoter, the growth on acetate was fully restored (Fig. 5a2), clearly demonstrating the essential role of the EMC pathway for acetate assimilation in *S. tsukubaensis*. In addition, this experiment confirmed the results of bioinformatics analysis, that *S. tsukubaensis* lacks functional alternative acetate-assimilating pathways, such as GLC.

**Fig. 6:**
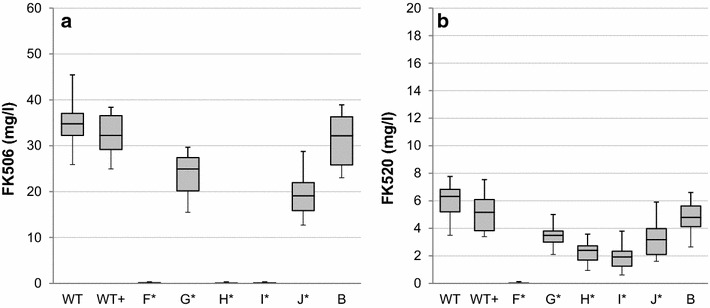
A comparison of FK506 (**a**) and FK520 (**b**) yields (mg/L) in engineered strains of *S. tsukubaensis*. *Bars* encompass 95 % of the sample population. *Horizontal lines* represent the median values, and *perpendicular lines* indicate extreme values (min, max). *Asterisks* represent statistically significant differences between different samples compared to control wild type samples (WT). WT: *S. tsukubaensis* NRRL 18488 (**a**); WT+: WT+ pSet152 (control strain with empty plasmid pSet152); *ΔallR* (**f**); *ΔallR* + *allR* (**g**); *ΔallR* + *ccr1* (**h**); *ΔallR* + *emc* (**i**); *ΔallR* + *allR_ecmOp* (**j**); *Δemc* (**b**) *Letters in brackets* correspond to the labels of the plasmids constructs presented on Fig. 4)

Notably, all efforts to complement the function of *ccr1* in the *Δemc* strain with the *allR* homologue have failed, since no growth of the strain *Δemc* + allR_ecmOp (Fig. 4e) was detected on the acetate-supplemented minimal medium NMMB (Fig. 5a3). Potential disturbances in the RNA transcription of the downstream genes of the *emc* operon, caused by exchange of *ccr1* in the chimeric *emc* operon with its homologue *allR* in the pABT42 plasmid (Fig. 4e), was ruled out by subsequent successful *in*-*trans* complementation of this strain with an additional copy of the *ccr1* gene. Finally, the further complemented strain *Δemc* + *allR_ecmOp* + *ccr1* (Fig. 4e1) entirely re-established the growth on acetate-supplemented minimal medium NMMB (Fig. 5a4). Functionality of the chimeric *emc* operon (strain *Δemc* + *allR_ecmOp*) was additionally demonstrated when the construct *allR_ecmOp* (Fig. 4j) was expressed in the *ΔallR* strain (Fig. 4f). The *ΔallR* is unable to produce either FK506 or FK520 (Fig. 6a, b); however, constitutive expression of the chimeric *emc* operon *allR_ecmOp* (Fig. 4j) completely re-established the production of both compounds (Fig. 6j), thus confirming functional *in trans* expression of *allR* in the chimeric operon. The inability of *allR* to take part in acetate assimilation is further demonstrated by the results of the qPCR analysis (Fig. 3a). These results show that the native *allR* gene is expressed in significant levels in the acetate-based NMMB medium; however, it does not support the functional EMC pathway. Altogether, these results show that *allR* cannot participate in the EMC pathway as a replacement for *ccr1*.

### Determining the role of the crotonyl-CoA reductase/carboxylase homologues *ccr1* and *allR* in FK506/FK520 biosynthesis

Complementation of the *S. tsukubaensisΔallR* deletion mutant (Fig. 4f) by the *allR* gene *in trans* under the constitutive promoter ermE* (Fig. 4g) re-established the production of both compounds, FK506 and FK520 (Fig. 6g). Interestingly, both compounds were also produced when the same deletion mutant (Fig. 4f) was complemented by the chimeric *emc* operon, containing *allR,* instead of *ccr1,* under the ermE* promoter (Fig. 4j). Nevertheless, the yield of FK506 was 25 % higher in the *ΔallR* + *allR* (Fig. 4g) strain, while FK520 was produced with a slightly higher yield by the *ΔallR* + *allR_ecmOp* (Fig. 4j) strain, with the chimeric *emc* operon (Fig. 6g, j). Overall, the ratio of FK520 in the mixture increased from around 15 %, when complemented with the *allR* gene alone, to 24 % in the strain expressing the chimeric operon *ΔallR* + *allR_ecmOp* (Fig. 4j). Considering that the chimeric operon re-established the production of both compounds, FK506 and FK520, it was reasonable to expect that the chimeric operon would also re-establish the growth of the*Δemc* strain (Fig. 4b) in minimal medium. Interestingly, this was not the case (Fig. 5a3), as discussed above.

Re-establishment of FK520 biosynthesis, but not of FK506 biosynthesis, by complementation of *S. tsukubaensisΔallR* strain (Fig. 4f) by the native *emc* operon, or just the *ccr1* gene alone, both expressed under the strong ermE* constitutive promoter was clearly demonstrated (Fig. 6h, i). As previously reported, under its native promoter, the *emc* operon does not enable significant production of FK520 in the *ΔallR* strain (Fig. 4f). This also agrees with the results obtained from the qPCR expression studies, where expression of *ccr1* in the *emc* operon under its native promoter was below the quantification limits in glucose/dextrin-based medium (Fig. 3b). Notably, expression of *ccr1* under the strong ermE* promoter, either alone (Fig. 4h) or in the context of the *emc operon* (Fig. 4j) in the *ΔallR* strain (Fig. 4f), is clearly not able to restore FK506 production (Fig. 6h, i), suggesting a narrow substrate specificity of Ccr1 for the 4-carbon substrate crotonyl-CoA.

## Discussion

To date, several pathways have been identified that provide ethylmalonyl-CoA, a common building block used in the biosynthesis of numerous polyketide natural products. As reviewed by Wilson and Moore [17], almost all *Streptomyces* strains that produce polyketides with ethyl side chain moieties in the polyketide backbone contain a *ccr* homologue in the corresponding gene cluster, this way ensuring a sufficient supply of the ethylmalonyl-CoA extender unit. For example, *ccr* homologues were identified in gene clusters encoding for the biosynthesis of the macrolide antibiotics tylosin [18], spiramycin [19], midecamycin [20], polyene elaiophylin [21] and of a number of other polyketides [4, 17]. Numerous *ccr* homologues have also been identified in other polyketide-producing strains, which are clearly involved in the provision of structurally unusual extender units [4].

On the other hand, Jung et al. [3] have recently demonstrated that genomes of many *Streptomyces* species encode for the EMC pathway, thus containing *ccr* homologues. Thus, the existence of multiple *ccr* homologues in a single genome, one located in the *emc operon*, and another one present in the gene cluster(s) encoding for the biosynthesis of secondary metabolites is a frequent phenomenon. However, not all gene clusters encoding for the biosynthesis of secondary metabolites with ethyl side chains contain a *ccr* homologue, as exemplified by the polyether antibiotics salinomycin and monensin, which are produced by *Streptomyces albus* and *Streptomyces cinnamonensis*, respectively [22–24]. In these two strains the *ccr* homologues involved in the EMC pathway most likely have additional roles in the provision of ethylmalonyl-CoA, which is incorporated into the polyketide backbones of these two polyether antibiotics [22]. In addition to the EMC pathway, it is also important to consider potential alternative sources of ethylmalonyl-CoA, such as β-oxidation of even-chain fatty acids and carboxylation of butyryl-CoA [25, 26].

We have identified two *ccr* homologues in the FK506 producing strain *S. tsukubaensis* NRRL 18488 (Fig. 1). One homologue, designated here as *ccr1,* is present in the *emc* operon and the second one is located in the FK506 gene cluster (designated here as *allR*) [11]. *S. tsukubaensis* thus represents a unique model system to study potential “cross-activity” of multiple *ccr* homologues, and its potential impact on primary metabolic pathways as well as on the production of FK506-related compounds. When considering metabolic/biosynthetic engineering approaches it is of significant importance to take into account the potential impact that “cross-activity” of multiple *ccr* gene homologues present in the same genome may have on the primary metabolic pathways, thus influencing the physiological properties of the microorganisms as well as the provision of building blocks for the biosynthesis of secondary metabolites.

The role of *all* genes, located on the left fringe of the FK506 biosynthetic cluster (designated also as *tcs* group of genes [9]), in providing allylmalonyl-CoA, is still not completely understood. Particularly the origin of ethylmalonyl-CoA, which could also be biosynthesised by the “All” proteins, is not known. It is possible that the main source of the undesired impurity FK520 in the FK506 producer *S. tsukubaensis* is a result of the promiscuous activity of the ALL enzyme complex, resulting in the simultaneous production of ethylmalonyl-CoA and allylmalonyl-CoA. Supporting this hypothesis, we demonstrated in our earlier work that the inactivation of *allR* located in the FK506 gene cluster completely abolished the production of both compounds, FK520 and FK506 [11]. However, in contrast to *S. tsukubaensis*, the inactivation of the *tcsC* homologue of the *ccr* gene (analogous to *allR*) in *Streptomyces sp.* KCTC 11604BP, which contains *all* (*tcs*) genes with 100 % identity at the nucleotide level [9], completely abolished the production of FK506, without affecting the biosynthesis of FK520 [9]; this suggests a different metabolic background for the biosynthesis of the ethylmalonyl-CoA extender unit in this strain. For example, expression of the *ccr1* gene present in the EMC pathway could be regulated differently in *Streptomyces sp.* KCTC 11604BP, similarly to what was demonstrated in previous studies with the regulators *fkbN* and *tcs7* [27, 28].

It was demonstrated [29] that the expression of the EMC pathway is generally induced in the presence of acetate, while it is not expressed in the presence of easily assimilable carbon sources, such as glucose [30, 31]. Therefore, the expression of the EMC pathway, that can potentially provide ethylmalonyl-CoA in the industrial media, likely depends on the medium composition. In this work we clearly demonstrate that *ccr1* (and likely the entire *emc* operon) is not expressed significantly in *S. tsukubaensis*, in the dextrin-rich industrial production medium PG3 (Fig. 3b). Accordingly, we did not observe a significant reduction (less than 10 %) in the production of FK520 following the disruption of the *ccr1* gene in *S. tsukubaensis* (Fig. 6b), in agreement with our earlier work [11]. Control experiments, where the cognate *ccr* homologues were used to re-constitute the disrupted EMC and ALL pathways resulted in successful re-establishment of FK520/FK506 production, simultaneously enabling growth in minimal medium (Figs. 5, 6). Importantly, these control experiments confirmed that the engineering approaches followed in order to construct the selected mutant strains were valid.

Our results show that introduction of the *ccr1* homologue from the EMC pathway into the *ΔallR* strain (Figs. 4f, 6f) resulted in the production of FK520, but not FK506, clearly demonstrating that Ccr1 can contribute to FK520 biosynthesis if expressed under a strong constitutive promoter. Importantly, this experiment also confirms that the Ccr1 can productively interact with the *all* proteins, thus replacing AllR activity. This result also suggests that Ccr1 is specific for the C4 substrate crotonyl-CoA but not for the C5 2-pentenoyl-CoA substrate. Therefore, it is possible that in some FK506 producing strains, such as the strain used by Mo et al. [9], Ccr1 may indeed influence the ethylmalonyl-CoA supply and the FK506/FK520 ratio, additionally depending on the composition of the medium used for the production of FK506. Thus, in addition to the expression profiles of the EMC and ALL pathways, substrate specificity and catalytic properties of *ccr*-homologues, Ccr1 and AllR, may also play an important role when considering “cross-activity” of both enzymes in primary/secondary metabolic pathways. It was demonstrated in the literature that substrate specificity of Ccr proteins can vary significantly, from selectivity for a single substrate to a broad specificity, as exemplified in the case of the *ccr*-homologue pteB from the *Streptomyces avermitilis* gene cluster [4, 32]. Accordingly, the complementation experiment where the *ΔallR* strain with overexpressed chimeric *emc* operon (Figs. 4j) in which the *ccr1* is replaced *in*-*frame* by *allR* resulted in production of FK506 and FK520, thus demonstrating relaxed specificity of AllR for C4 and C5 substrates (Fig. 6j). Surprisingly, when the same construct was overexpressed in the *ccr1*-inactivated strain (Fig. 4e) the chimeric *emc* operon did not re-establish the capacity of this strain to grow on minimal medium containing acetate as the sole carbon source (Fig. 5a3). Considering the relaxed specificity of AllR for C4 and C5 substrates, and the very high degree of similarity between the amino acid sequences of Ccr1 and AllR (Additional file 2: Figure S2) this is a somewhat surprising result. It is likely that the chimeric *emc* operon containing *allR* does not provide a sufficient flow of metabolites through the EMC pathway. In contrast, additional complementation of the strain with the chimeric *emc* operon using *in trans* complementation with the *ccr1* gene expressed under strong constitutive promoter (*Δemc* + *allR_ecmOp* + *ccr1,* Fig. 4e1) re-established growth on acetate (Fig. 5a4), confirming that the genes downstream of *allR* in the chimeric *emc* operon are sufficiently expressed; therefore, the inability of the strain *Δemc* + *allR_ecmOp* + *ccr1,* with a chimeric operon, to grow in minimal medium supplemented with acetate is likely related to the enzymatic properties of AllR, such as e.g. lower affinity (higher K_M_) of AllR for the crotonyl-CoA substrate, compared to Ccr1.

Finally, it is also important to consider potential differences in the enzymatic mechanisms of the EMC and ALL pathways, as discussed by Mo et al. [9] and recently by Jiang et al. [33]. Intermediates of the EMC pathway are CoA-activated. In contrast, it was suggested that the biosynthesis of allylmalonyl and potentially also ethylmalonyl extender units by the ALL enzymes is likely carried out on ACP-bound intermediates, although CoA-activated substrates cannot be ruled out [33]. Thus, even though the *emc* operon is expressed, the CoA-bound ethylmalonyl extender unit might not be loaded efficiently on ALL enzymes. Further detailed biochemical and functional analyses of different *ccr* homologues will be necessary in the future. It is likely that in addition to differences in the regulation of the expression of homologues involved in the EMC pathway or polyketide biosynthesis, different enzymatic properties may have evolved in the two groups of homologous enzymes to optimally fulfil their roles in the corresponding metabolic/biosynthetic pathway.

Thus, on the one hand, their different functional role is based on differential regulation of gene expression strongly influenced by medium composition, and on the other hand it is related to the different enzymatic properties, such as affinity for the substrate.

We have therefore demonstrated that, despite being evolutionarily closely related, the functional roles of these two *ccr* gene homologues in *S. tsukubaensis* are clearly separated between primary and secondary metabolic pathways, with minimal cross-activity.

## Conclusions

In conclusion, we have provided an understanding of the functional roles of two homologous genes, *ccr1* and *allR*, in the FK506-producing strain *S. tsukubaensis* NRRL 18488, the progenitor of high producing strains used in the industrial production of FK506 [34, 35]. Our results show that the *ccr1* gene, that is part of the EMC pathway, does not have a significant role in the biosynthesis of FK506 or the structurally related impurity FK520 when cultivated on carbohydrate-based media. Thus, media composition such as carbon and nitrogen sources used for cultivation may have a significant influence on the expression of the *emc* operon and *all* genes. When overexpressed under the control of a strong constitutive promoter, the *ccr1* gene can take part in the biosynthesis of ethylmalonyl-CoA and thereby FK520, but not of the main product FK506. In contrast, even when constitutively expressed, the *allR* gene, part of the FK506 biosynthetic cluster, is not able to replace *ccr1* in establishing a functional EMC pathway and thereby support growth on acetate as the sole carbon source which is surprising, considering the broad specificity of AllR for C4 and C5 substrates. Thus, different regulation of the expression of both genes, and corresponding pathways EMC and ALL, in combination with the catalytic properties of Ccr1 and AllR enzymes, most likely determine the exclusive role of *ccr1* in the primary metabolism, and *allR* in the secondary metabolic pathway, such as the biosynthesis of FK506/FK520. A similar situation might be encountered in the future in other actinomycete strains containing more than one *ccr* homologue in their genomes.

## Methods/experimental procedures

### Bacterial strains, medium composition and cultivation conditions

We based our studies on FK506 and FK520-producing organism *Streptomyces tsukubaensis* NRRL 18488, *S. tsukubaensis* Δ*allR* in-frame deletion mutant strain [11] and *S. tsukubaensis* Δ*ccr1* with a thiostrepton resistance cassette disrupting the first open reading frame of the operon for ethylmalonyl-CoA metabolic pathway [11] containing genes *ccr1, ecm, mml, mch* and *mcd* (Additional file 1: Table S1).

### Microbiological methods

The spores and seed culture were prepared as described before [5]. The ISP4 medium [16] was used for spore stock preparation. Apramycin (50 mg/mL), kanamycin (25 mg/mL) and thiostrepton (25 mg/mL) were added to the solid and liquid medium after sterilisation, as required. For the purpose of mRNA isolation and for detection of FK506 and FK520 production liquid cultures spores of *S. tsukubaensis* strains (1 % v/v) were inoculated in seed medium VG3 and incubated at 28 °C and 220 rpm for 24–48 h. 10 % (v/v) of seed culture was used for the inoculation of production medium PG3 [5]. Cultivation was carried out at 28 °C, 220 rpm for 6 days. Detection and quantification of FK506 and FK520 with HPLC analysis was performed as described previously [5]. For the *S. tsukubaensis* assimilation/utilisation studies of acetate as sole carbon source minimal medium NMMB [36] with sodium acetate (2 g/l) was used [37].

### Construction of plasmids and mutant strains

Standard general methods for manipulation of DNA were carried out as described by Sambrook and Russel [38] and [36]. Genomic DNA for sequencing and PCR amplification was prepared using standard procedures [38]. Plasmid vectors were propagated in *E. coli* DH10β grown in 2TY medium [38]. *S. tsukubaensis* transformation was carried out using *E. coli*-*Streptomyces* conjugation procedure with *E. coli* ET12567 containing the conjugation facilitating plasmid pUZ8002 [39]. The PCR fragments were initially cloned into pUC19 and their DNA sequence confirmed by sequencing. Further, the selected DNA fragments were excised from pUC19 using NdeI and XbaI restriction enzymes, gel purified and subcloned into the ΦC31-based integrative expression vector pSET152 [40], containing a relatively strong constitutive ermE* promoter and a *Streptomyces* ribosome binding site [41].

### Overexpression of the *ccr1* gene homologue from EMC pathway of *S. tsukubaensis*

For the purpose of overexpressing the *ccr1* gene, the 1473 bp fragment, which incorporates the *ccr1* gene, was amplified. The primers for PCR amplification were designed as follows: ccr1F primer containing an NdeI restriction site and ccr1R containing an XbaI restriction site. Further, the fragment was digested with NdeI and XbaI restriction enzymes and ligated into a previously linearized vector pSET152 + P_ermE*_ with corresponding restriction enzymes. After sequence confirmation, the plasmid construct pSET152 + P_ermE*_ + *ccr1* was then conjugated in *S. tsukubaensis* strain Δ*allR*.

### Overexpression of *allR* gene from FK506 gene cluster of *S. tsukubaensis*

Primers for the purpose of overexpressing the *allR* gene were designed as follows: allR-F primer with an NdeI restriction site and ccr2-R with an XbaI restriction site. The 1335 bp long fragment was PCR amplified, identified via gel electrophoresis and purified. The 5′-ends of the fragment were phosphorylated using the T4 polynucleotide kinase. The fragment was ligated into pUC19 vector, previously linearized with restriction enzyme NdeI, cohesive ends were filled with the DNA polymerase I Klenow fragment (Fermentas) and dephosphorylated with alkaline phosphatase. Before continuing, we confirmed the nucleotide sequence of the cloned PCR fragment by sequencing. The *allR* gene was then excised with using partial restriction with NdeI and XbaI enzymes, gel-purified and ligated into pSET152 + P_ermE*_, previously linearized using the restriction enzymes NdeI and XbaI. The construct pSET152 + P_ermE*_ + *allR* was introduced into electrocompetent *E. coli* strain ET12567/pUZ8002 and then conjugated into *S. tsukubaensis ΔallR*.

### Overexpression of *emc operon*

For the purpose of complementation of *emc* operon in strain *S. tsukubaensis* Δ*ccr1* we designed primer pair Ccr1ExpF/ecmOpR. Using genomic DNA of *S.tsukubaensis* WT strain as template we amplified entire 6067 bp long operon containing *ccr1, ecm, mml, mch* and *mcd* genes. After the sequence confirmation operon was cloned into a pSET152-based plasmid under control of constitutive ermE* promoter and ribosome binding site as described before [40].

### Construction of chimeric *emc* operon *allR_ecmOp* in which *ccr1* is replaced by *allR*

For complementation studies, the *emc* operon from *S. tsukubaensis* was cloned into the pSET152-based integrative vector containing ΦC31-phage integrase as described by Magdevska et al. ([40], (Fig. 4, Additional file 3: Table S2) and expressed under relatively strong constitutive promoter ermE* [41] designated as pABT2 (Additional file 3: Table S2). We have constructed integrative plasmid pABT42 where the *ccr1* gene from *S. tsukubaensisemc* operon was replaced *in frame* by *allR*, resulting in a chimeric *emc* operon (Fig. 4e), thus allowing transcription of downstream genes. Functional expression of the downstream genes was confirmed by further *in*-*trans* complementation of this strain by *ccr1* using plasmid pABT10 (Fig. 4e1, Additional file 3: Table S2). Constitutive expression of *ccr1* or *allR* individually was achieved by constructing pABT3 and pABT4 vectors, respectively, and by *in*-*trans* complementation using ΦC31-based phage integrase vector expressed under the constitutive ermE* promoter (Additional file 3: Table S2). Considering that *ccr* homologues *ccr1* and *allR* display high similarity at the amino acid level (Additional file 2: Figure S2), it was not difficult to construct the pABT42 plasmid for *in*-*frame* replacement of *ccr1* with *allR*, which upon transformation into the *Δemc* strain resulted in the *Δemc* + *allR_ecmOp* strain (Fig. 4e).

In detail, we separately amplified the two segments of the new operon: the *allR* gene and the *emc* operon (*ecm*, *mml*, *mch* and *mcd* gene) without the *ccr1* gene at the 5′-end. To amplify the 1335 bp long region with the *allR* gene, we used primers Erm-ccr2-F and ccr2-ecmOpV3-R. To amplify the 4835 bp long region of the second segment, we used primers ccr2-ecmOpV3-F and ecmOp-pSet-R. All primers were designed to contain 15–20 bp of overlapping homologous DNA regions (Additional file 3: Table S3). In the first segment, containing the *allR* gene, the primers overlap with the pSET152 + P_ermE*_ vector on the left side, and the beginning of the *ecm* gene on the right side. Primers for the second segment were designed to overlap with the *allR* gene on the left, and pSET152 + P_ermE*_ vector on the right side. PCR fragments were identified via gel electrophoresis using the DNA size standard. Fragments were gel-purified and ligated into pSET152 + P_ermE*_ vector, using the commercial Gibson assembly cloning kit (New England Biolabs). Prior to ligation, we linearized the vector with restriction enzymes NdeI and XbaI. The construct pSET152 + P_ermE*_ + *allR_ecmOp* was then transformed into the *E. coli* strain DH10β, and after plasmid isolation, verified by sequencing. Confirmed construct was then introduced into electrocompetent *E. coli* strain ET12567/pUZ8002 and then conjugated into *S. tsukubaensis* strains *Δemc* and *ΔallR*.

### Overexpression of *ccr1* gene in strain with chimeric *emc* operon *allR_ecmOp*

The *ccr1* gene was excised from the vector pSET152 + P_ermE*_ + *ccr1* together with the ermE* promotor region, using restriction enzymes EcoRI and XbaI. The 1649 bp long fragment was gel purified and cloned into integrative plasmid pSok804, previously linearized using EcoRI and XbaI. pSok804 is a site-specific integrative plasmid, which stably incorporates into *Streptomyces* genome at attB^VWB^ site, due to the integrase gene from bacteriophage VWB, carried on plasmid [42]. Importantly the integration into the VWB integration site present in *S. tsukubaensis* NRRL 18488 genome is compatible with simultaneous stable integration into a different att site of the pSet152 plasmid, which uses the bacteriophage ΦC31 integrase [6]. Kanamycin-resistance cassette (1323 bp) was additionally introduced into pSok804 plasmid, for the purpose of concurrent integration with pSet152-based plasmids. The plasmid pSok804 Kn P_ermE*_ + *ccr1* was constructed using the modified pSok804, confirmed by sequencing, and further conjugated in *S. tsukubaensis* strains *Δemc* and *ΔallR*.

### Gene expression analysis by qPCR

Expression of *allR* and *ccr1* genes in WT strain was monitored in the time course of the fermentation process production medium (PG3) and in NMMB medium supplemented with sodium acetate (2 g/l). Total RNA from mycelia was extracted after 36, 72 and 103 h of fermentation.

As described previously, a seed culture was used for the inoculation of 250-ml Erlenmeyer flasks containing 50 ml of PG3 production medium [27], and cultivation was carried out at 28 °C at 220 rpm for 62 h. A 2-ml sample was collected and mixed thoroughly with 4 ml of RNAlater RNA stabilization reagent (Qiagen). After 5 min of incubation at room temperature, the mixture was stored at −80 °C. Primers and probes for *allR*, *ccr1* and *hrdB* gene (Additional file 3: Table S4) were designed as Custom TaqMan Gene expression Assays (Life Technologies). RNA was isolated, DNase treated and reverse transcribed and qPCR was set as described earlier [43]. The standard curve method was used for relative gene expression quantification, and the transcript accumulation of monitored genes was normalized to the expression of 16S rRNA and *hrdB* [44] reference genes.

### Statistical analysis

For FK506/FK520 measurements, at least three independent colonies (transformants) were tested for each engineered strain in the three consecutive independent experiments. Each independent colony (transformant) was tested in duplicates. For qPCR two independent colonies of the wild type strain were used, each with two experimental replicates to confirm reproducibility. Yields of FK506/FK520 were calculated with SAS/STAT software using means and the univariate procedure to test the normality of distribution. Using the GLM model, data were calculated as least mean square and are presented as an average change observed from all experiments when comparing least mean square values to the wild-type control least mean square value of each experiment.

## Additional files

10.1186/s12934-015-0352-z The *emc* operon from *S. tsukubaensis*. **Table S1.** Description of the genes located in the emc operon from *S. tsukubaensis* NRRL 18488.
10.1186/s12934-015-0352-z Protein sequence alignment of Ccr family of proteins, Ccr1 and AllR (ClustalW2).
10.1186/s12934-015-0352-z Strains and plasmids used in this study. **Table S3.** Primers used in this study. **Table S4.** Primers for qPCR.

## Abbreviations

bp: base pair
WT: Wild type
EMC: ethylmalonyl-CoA pathway
*ccr*: crotonyl-CoA carboxylase/reductase
ALL: operon providing an unusual extender unit allylmalonyl-CoA
PKS: polyketide synthase
NRPS: non-ribosomal peptide synthetase
GLC: glyoxylate cycle
SNAC: N-acetylcysteamine
*ecm*: ethylmalonyl-CoA mutase
*mml*: β-methylmalonyl-CoA/L-malyl-CoA lyase
*mch*: mesaconyl-CoA hydratase
*mcd*: methylsuccinyl-CoA dehydrogenase
PCR: polymerase chain reaction
qPCR: quantitative real-time PCR
