# Supplementary material for: Roles of the crotonyl-CoA carboxylase/reductase homologues in acetate assimilation and biosynthesis of immunosuppressant FK506 in Streptomyces tsukubaensis
Source: Microb Cell Fact. 2015 Oct 14;14:164. doi: 10.1186/s12934-015-0352-z (PMC4606968; doi:10.1186/s12934-015-0352-z)
Supplement: Supplementary file 1 — 10.1186/s12934-015-0352-z The emc operon from S. tsukubaensis. Table S1. Description of the genes located in the emc operon from S. tsukubaensis NRRL 18488. [file 12934_2015_352_MOESM1_ESM.docx]

**Additional file 1**


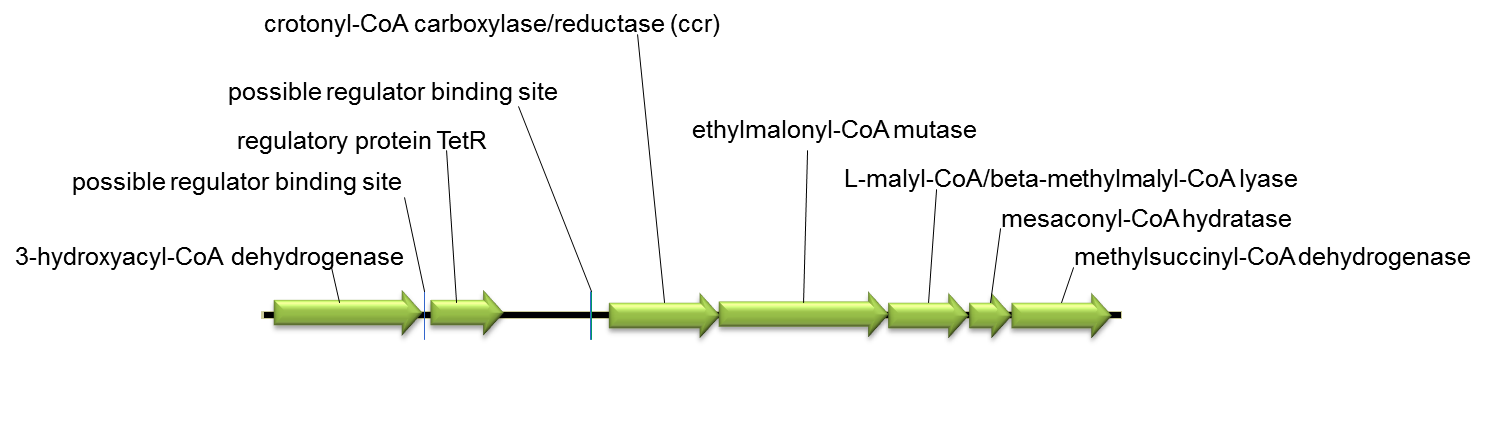


**Figure S1**. The *emc* operon from *S. tsukubaensis*

**Table S1**. Description of the genes located in the emc operon from *S. tsukubaensis* NRRL 18488

| **Gene** | **Gene ID** | **Locus tag** | **Proposed function** | **Length**  **(amino acids)** |
| --- | --- | --- | --- | --- |
| *hcd* | GI:385670475 | STSU_05033 | 3-hydroxyacyl-CoA dehydrogenase | 511 |
| *tetR* | GI:385670476 | STSU_05038 | putative TetR family  regulatory protein | 292 |
| *ccr1* | GI:385670467 | STSU_05043 | crotonyl-CoA carboxylase/  reductase | 445 |
| *ecm* | GI:385670468 | STSU_05048 | ethylmalonyl-CoA mutase | 681 |
| *mml* | GI:385670469 | STSU_05053 | β-methylmalyl-CoA lyase | 323 |
| *mch* | GI:385670470 | STSU_05058 | mesaconyl-CoA hydratase | 171 |
| *msd* | GI:385670471 | STSU_05063 | methylsuccinyl-CoA dehydrogenase | 401 |
