# Supplementary material for: Roles of the crotonyl-CoA carboxylase/reductase homologues in acetate assimilation and biosynthesis of immunosuppressant FK506 in Streptomyces tsukubaensis
Source: Microb Cell Fact. 2015 Oct 14;14:164. doi: 10.1186/s12934-015-0352-z (PMC4606968; doi:10.1186/s12934-015-0352-z)
Supplement: Supplementary file 2 — 10.1186/s12934-015-0352-z Protein sequence alignment of Ccr family of proteins, Ccr1 and AllR (ClustalW2). [file 12934_2015_352_MOESM2_ESM.docx]

**Additional file 2**

**Figure S2**. Protein sequence alignment of Ccr family of proteins, Ccr1 and AllR (ClustalW2)

Amino acid ligament of *ccr1* and *allR* gene homologues from EMC and FK506 gene cluster, respectively. A key amino acid residues involved in the NADPH binding site are labelled in blue. Amino acid residues likely influencing substrate specificity of the *ccr* homologues based on the literature data are labelled in red.
