# Supplementary material for: Roles of the crotonyl-CoA carboxylase/reductase homologues in acetate assimilation and biosynthesis of immunosuppressant FK506 in Streptomyces tsukubaensis
Source: Microb Cell Fact. 2015 Oct 14;14:164. doi: 10.1186/s12934-015-0352-z (PMC4606968; doi:10.1186/s12934-015-0352-z)
Supplement: Supplementary file 3 — 10.1186/s12934-015-0352-z Strains and plasmids used in this study. Table S3. Primers used in this study. Table S4. Primers for qPCR. [file 12934_2015_352_MOESM3_ESM.docx]

**Additional file 3**

**Table S2**: Strains and plasmids used in this study

| **Strain/vector name** | **Description** | **Promoter** | **Gene** | **Reference** |
| --- | --- | --- | --- | --- |
| WT | *S. tsukubaensis* NRRL 18488 |  |  |  |
| WT pSET152 | Control; ɸC31, apramycin^R^ |  |  | [[1](#_ENREF_1)] |
| Δ*emc* | *emc* operon inactivation with Ts^R^ cassette |  |  | [[1](#_ENREF_1)] |
| Δ*emc* + *ccr1* | ɸC31, apramycin^R^ | P_ermE*_ | *ccr1* | This work |
| Δ*emc* + *emc* | ɸC31, apramycin^R^ | P_ermE*_ | *ccr1*, *ecm*, *mml*, *mch*, *mcd* | This work |
| Δ*emc* + *allR_ecmOp* | ɸC31, apramycin^R^ | P_ermE*_ | *allR*, *ecm*, *mml*, *mch*, *mcd* | This work |
| Δ*emc* + *allR_ecmOp + ccr1* | ɸC31, apramycin^R^;  VWB; kanamycin^R^ | P_ermE*_ | *allR*, *ecm*, *mml*, *mch*, *mcd; ccr1* | This work |
| Δ*allR* | *allR* in frame deletion |  |  | [[1](#_ENREF_1)] |
| Δ*allR* + *allR* | ɸC31, apramycin^R^ | P_ermE*_ | *allR* | This work |
| Δ*allR + ccr1* | ɸC31, apramycin^R^ | P_ermE*_ | *ccr1* | This work |
| Δ*allR* + *emc* | ɸC31, apramycin^R^ | P_ermE*_ | *ccr1*, *ecm*, *mml*, *mch*, *mcd* | This work |
| Δ*allR* + *allR_ecmOp* | ɸC31, apramycin^R^ | P_ermE*_ | *allR*, *ecm*, *mml*, *mch*, *mcd* | This work |
| pABT2 | pSet152 based vector;  ɸC31, apramycin^R^ | P_ermE*_ | *ccr1*, *ecm*, *mml*, *mch*, *mcd* | This work |
| pABT3 | pSet152 based vector;  ɸC31, apramycin^R^ | P_ermE*_ | *ccr1* | This work |
| pABT4 | pSet152 basedvector;  ɸC31, apramycin^R^ | P_ermE*_ | *allR* | This work |
| pABT10 | pSok804 based vector;  VWB, kanamycin^R^ | P_ermE*_ | *ccr1* | This work |
| pABT42 | pSet152 based vector;  ɸC31, apramycin^R^ | P_ermE*_ | *allR*, *ecm*, *mml*, *mch*, *mcd* | This work |

**Table S3**: Primers used in this study

|  |  |  |
| --- | --- | --- |
| *allR* | allR-F:  5'–ACATATGACCCACGTTCGCGACGCCG-3' NdeI | Ccr2-R:  5'–AAAATCTAGATCACCGGGGCTGCCCCTTCCG GAACA-3' XbaI |
| *ccr1* | ccr1F:  5'-AACATATGAAGGAAATCCTGGACGCGA TCTC–3' NdeI | ccr1R:  5'-TTTCTAGATTGCGCCGGTACAGCTCGTTC–3' XbaI |
| *emc* | Ccr1ExpF:  5'-AACATATGAAGGA AATCCTGGACGCGATCTC–3' NdeI | ecmOpR:  5'- ATCTAGAATCAACCCTGGATTCGGTACTCCTCC–3' XbaI |
| artificial operon *allR_ecmOp* 1 part | Erm-ccr2-F:  5'-GGAGGACCCCACATATGATGACCCAC GTTCGCGACG–3' | ccr2-ecmOpV3-R  5'-TCCCGCACACCCATGCCCTCACCGGGGCTGCCCCTT–3' |
| artificial operon *allR_ecmOp* 2 part | ccr2-ecmOpV3-F  5'-GCAGCCCCGGTGAGGGCATGGGTGTG CGGGA CGAGGA–3' | ecmOp-pSet-R  5'-TGCAGGTCGACTCTAGATCAACCCTGGATTCGG TACTCCT–3' |

**Table S4**: Primers for qPCR

| **Assay name** | **Target sequence** | **Primer/probe sequence** | | **Assay efficiency** |
| --- | --- | --- | --- | --- |
| hrdB | GI:385669860 | F: | TGAAGCAGATCGGCAAGGT | 101% |
|  |  | R: | GAAGAGACCGGCCTCGAT |  |
|  |  | P: | FAM-CCCCTCCTCAACGCC-MGB |  |
| ccr1 | GI:385670467 | F: | CGCAGTCCCCGAGTCCTA | 96% |
|  |  | R: | CGACATGCAGCGACTTACG |  |
|  |  | P: | FAM-CTCGGCTTCGTCCTTG-MGB |  |
| allR | GI:385664383 | F: | GGGCCGCCGTTGTC | 99% |
|  |  | R: | CTGGACCTGGACCGTCTTG |  |
|  |  | P: | FAM-CCGACCACCAGGCACT-MGB |  |
| 16s rRNA | Kirm et al, 2013 | | | 94% |

1. Kosec G, Goranovič D, Mrak P, Fujs Š, Kuščer E, Horvat J, Kopitar G, Petković H: **Novel chemobiosynthetic approach for exclusive production of FK506.** *Metab Eng* 2012, **14:**39-46.
